# Facial Edema After Nuclear Stress Test

**DOI:** 10.7759/cureus.76780

**Published:** 2025-01-02

**Authors:** Emily Saurborn, Danielle Roth, Shane Cook

**Affiliations:** 1 Dermatology, Marshall University Joan C. Edwards School of Medicine, Huntington, USA

**Keywords:** angioedema, dermatologic presentation, drug-related side effects and adverse reactions, nuclear stress test, radiotracer

## Abstract

Angioedema involves fluid accumulation into the interstitial spaces of the dermis, subcutaneous tissue, and mucosal surfaces. While usually benign and self-limited, angioedema can lead to laryngeal edema, a life-threatening condition. The most common causes are histamine-mediated allergic reactions. However, angioedema can also be mediated by bradykinin. Bradykinin-mediated angioedema can occur in the setting of hereditary deficiency of C1q esterase and after exposure to several medications. Drug-induced angioedema is most commonly a secondary complication of non-steroidal anti-inflammatory drug (NSAID) or sulfa drug use. All providers must be aware of the potential side effects of the medications they use or prescribe. We present a case of angioedema resulting from the use of technetium-99m sestamibi tracer injection during an adenosine nuclear stress test.

## Introduction

Angioedema is a self-limiting condition due to immune dysfunction resulting in edema most commonly affecting the face or genital region but may affect any body part. This condition may be a result of a hereditary or an acquired disorder resulting from mast cell degranulation or a bradykinin-mediated reaction [1]. Bradykinin-induced angioedema is a result of either a hereditary or an acquired enzyme deficiency. The C1q esterase inhibitor deficiency results in the inability of the serine proteases to activate, causing impaired cleavage of the complement cascade and resultant buildup of bradykinin [2]. Potential causes of a C1q esterase deficiency may include lymphoproliferative disorders or autoimmune diseases [3]. Mast cells releasing histamine leading to angioedema may result from an immune-mediated reaction from medication or allergens, whereas bradykinin-induced angioedema is not mediated by immunoglobulin E (IgE) antibodies [4,5]. The most common drugs to cause histamine-mediated angioedema are sulfa drugs and non-steroidal anti-inflammatory drugs (NSAIDs) [4]. NSAIDs may also contribute to the development of angioedema through alterations in the arachidonic acid pathway, shunting more metabolites into the 5-lipoxygenase pathway, increasing the production of leukotriene [6]. 

Patients normally present with rapid swelling of the dermis, subcutaneous tissues, and mucosal surfaces alongside possible urticaria [2,7]. A serious complication of angioedema is laryngeal edema, which can result in respiratory compromise [1]. Bronchospasm and urticaria are more commonly associated with a drug-induced reaction, while laryngeal edema is commonly seen in both hereditary and acquired cases [1]. It is important to recognize the presentation of this diagnosis, to initiate early treatment, and to advise the patient to avoid any potential triggers.

## Case presentation

A 46-year-old woman with a past medical history of hyperactive airway disorder presented to Dermatology with a 24-hour history of facial redness, swelling, and tenderness. The patient was referred by Internal Medicine after being worked up the previous day for facial cellulitis and started on amoxicillin-clavulanate without any improvement. The patient denied trauma, recent medication changes, or a history of a similar reaction. Vitals were stable. Physical exam revealed swelling and tenderness localized to the forehead, periorbital skin, and temples without laryngeal involvement, consistent with angioedema, and was otherwise unremarkable. Further workup included a complete blood count, comprehensive metabolic panel, C4 levels, and computed tomography (CT) of the head to rule out orbital cellulitis. Labs revealed an elevated C4 level consistent with drug-induced angioedema though no association was found upon review of medications. The patient was prescribed fexofenadine in addition to continuing the antibiotic regimen with follow-up the next day.

At follow-up with Dermatology, increased edema was noted, while airways remained clear. At that time, it was discovered that the patient had an injection of technetium-99m (Tc-99m) sestamibi tracer during an adenosine nuclear stress test the day preceding her symptoms. Drug-induced angioedema due to Tc-99m sestamibi tracer was suspected, which was consistent with her elevated C4. A prednisone taper consisting of 60 mg daily for three days, 40 mg daily for four days, and 20 mg daily for 10 days was started in addition to continuing fexofenadine. The patient was prescribed an EpiPen and instructed to go to the emergency department if her symptoms progressed to involve her airways.

## Discussion

Angioedema involves fluid leakage into the interstitial space resulting from an overactivation of the immune system, either histamine-induced or bradykinin-induced. Histamine-induced angioedema results from increased vascular permeability as a result of histamine release from mast cells or basophils. Bradykinin-induced angioedema results from increased bradykinin, a vasodilator, that contributes to vascular permeability. C1q esterase, a regulator protein, inhibits the activation of the kinin-kallikrein system, inhibiting the overproduction of bradykinin [8]. Enzyme deficiency will lead to elevated levels of bradykinin, predisposing patients to an increased risk of angioedema. Factors such as genetics, prolonged medication use, allergic reactions, and radiotracers can trigger such reactions [9]. Radionuclide tracers are useful in the evaluation of myocardial function in patients with known or suspected coronary artery disease. Compared to traditional alternatives such as advanced CT, magnetic resonance imaging, or enhanced X-rays, radiotracers are considered safer due to the small amount injected and lower frequency of administration [10]. However, adverse reactions can still occur, with the most life-threatening reactions consisting of allergic reactions [9]. In the package insert of Cardiolite (Tc-99m sestamibi), there is a warning for angioedema with a prevalence rate of 0.5% [11,12]. 

Angioedema secondary to Tc-99m sestamibi injection is a rare adverse reaction with only one other reported case in the literature. Makaryus et al. identified a 63-year-old woman who underwent a routine nuclear testing and, within 24 hours after the completion of the test, presented with severe angioedema [13]. The patient reportedly had profound swelling of the tongue with drooling and difficulty swallowing that improved with intravenous steroids and antihistamines. While the patient was on an angiotensin-converting enzyme (ACE) inhibitor, she had taken the medicine for years without any adverse effects and did not take the medication the day of the nuclear stress test. This, coupled with the temporality of events, led the authors to conclude the patient's angioedema was caused by the Tc-99m sestamibi tracer [13].

Although angioedema is a rare adverse reaction, there is evidence to suggest that other allergic reactions such as erythroderma, anaphylaxis, and erythema multiforme are consistent with an injection of Tc-99m sestamibi. Doukaki et al. described a 71-year-old man who presented with erythroderma affecting greater than 90% of his body surface following a nuclear radiotracer injection of Tc-99m sestamibi [14]. Additionally, Mujtaba et al. reported an anaphylactic reaction occurring during the first injection of Tc-99m sestamibi radiotracer [15]. Lastly, Thomson et al. concluded that their patient who had developed erythema multiforme 48 hours post-injection had an allergic reaction to Tc-99m sestamibi [16]. 

## Conclusions

Although adverse effects are rare, there is still evidence to suggest that allergic reactions can occur after the injection of Tc-99m sestamibi tracer. Our patient's symptoms are consistent with other literature findings regarding the use of Tc-99m sestamibi. Due to the nuclear chemical stress test and rapid onset of facial edema in the absence of signs of infection, it is believed to be that the radiotracer used in the nuclear chemical stress test induced the angioedema in this patient. It is important to observe the patient's symptoms and monitor the progression, to implement early treatment, and to prevent airway closure. In this patient who recovered, it is important to advise against radionuclide tests in the near future.
